# A European survey on allogeneic haematopoietic cell transplantation for myelofibrosis on behalf of the Chronic Malignancies Working Party of the EBMT: focus on ‘real world’ experience of JAK inhibitors, splenomegaly management and novel agents in the transplant algorithm

**DOI:** 10.1038/s41409-025-02780-2

**Published:** 2025-12-24

**Authors:** Alexandros Rampotas, Jose Maria Aspa-Cilleruelo, Linda Koster, Daniele Avenoso, Jakob Passweg, Elisa Sala, Marie Robin, Anders Eivind Myhre, Moniek de Witte, Erfan Nur, Patrice Chevallier, Thomas Schroeder, Micha Srour, Patrizia Chiusolo, Urpu Salmenniemi, Mareike Verbeek, Maria Chiara Finazzi, Cristina Castilla-Llorente, Marie Therese Rubio, Patryk Sobieralski, Katja Sockel, Ahmad Alabdulkarim, Joanna Drozd-Sokolowska, Kavita Raj, Giorgia Battipaglia, Tomasz Czerw, Nicola Polverelli, Juan Carlos Hernández-Boluda, Donal P. McLornan

**Affiliations:** 1 Department of Haematology, University College London, London, UK; 2 Department of Haematology, UCL Cancer Institute, London, UK; 3 Sheffield Teaching Hospitals NHS Foundation Trust, Sheffield, UK; 4 EBMT Leiden Study Unit, Leiden, The Netherlands; 5 Unit of Blood Diseases and Bone Marrow Transplantation, Department of Clinical and Experimental Science, University of Brescia, ASST Spedali Civili di Brescia, Brescia, Italy; 6 Basel University Hospital, Basel, Switzerland; 7 University Hospital Ulm, Ulm, Germany; 8 Service d’hématologie-greffe, Hôpital Saint-Louis, APHP, Université de Paris Cité, Paris, France; 9 Department of Haematology, Oslo University Hospital, Oslo, Norway; 10 UMC Utrecht, Utrecht, The Netherlands; 11 Amsterdam UMC, University of Amsterdam, Amsterdam, The Netherlands; 12 Hematology Department, Nante University Hospital, Nantes, France; 13 University Hospital Essen, Essen, Germany; 14 CHU Lille, Lille, France; 15 Fondazione Policlinico Universitario A. Gemelli-IRCCS, Rome, Italy; 16 Helsinki University Hospital, Helsinki, Finland; 17 TUM University Hospital, Munich, Germany; 18 ASST Papa Giovanni XXIII, Bergamo, Italy; 19 Gustave Roussy Cancer Campus, Villejuif, France; 20 Nancy Hospital, Vandoeuvre les Nancy, Nancy, France; 21 Department of Hematology and Transplantology, Medical University of Gdańsk, University Clinical Center, Gdańsk, Poland; 22 University Hospital Dresden, Dresden, Germany; 23 University Clinical Centre, Medical University of Warsaw, Warsaw, Poland; 24 Hematology Department, Federico II University of Naples, Naples, Italy; 25 Maria Skłodowska-Curie National Research Institute of Oncology, Gliwice Branch, Poland; 26 Unit of Bone Marrow Transplantation and Cellular Therapies, Fondazione IRCCS Policlinico San Matteo, Pavia, Italy; 27 Department of Hematology, Hospital Clínico Universitario, Valencia, Spain

**Keywords:** Myeloproliferative disease, Health services

## Abstract

Allogeneic haematopoietic cell transplantation (allo-HCT) remains the only potentially curative option for patients with myelofibrosis (MF), yet the integration of JAK inhibitors (JAKi) and novel agents into transplant pathways has created increasing complexity. To capture current real-world practice, the EBMT Chronic Malignancies Working Party conducted a survey of 19 high-volume European centres performing MF allo-HCT. Most centres (68%) routinely initiated JAKi, primarily ruxolitinib, in transplant-eligible patients prior to conditioning, with goals of splenomegaly reduction and symptom control. Management of ruxolitinib intolerance or resistance was heterogeneous, with strategies including switching to alternative JAKi, proceeding directly to allo-HCT, or enroling in clinical trials. Peri-transplant approaches also varied: over half of centres continued ruxolitinib throughout conditioning, while others employed tapering or abrupt discontinuation. Experience with newer JAKi and investigational therapies was limited. Post-transplant, most centres did not routinely reintroduce JAKi, although some used them for relapse or GVHD mitigation. Notably, many centres reported transplant delays due to prolonged medical therapy, with adverse consequences including disease progression. These findings highlight significant heterogeneity in practice, which is likely to increase as more novel agents are integrated in treatment algorithms. Harmonised, multidisciplinary guidelines to optimise timing and outcomes for MF patients eligible for allo-HCT are needed.

## Introduction

Myelofibrosis (MF) is a markedly heterogeneous Myeloproliferative Neoplasm (MPN), with disease trajectories ranging from an initial, relatively indolent course, through to rapidly progressive forms with bulky splenomegaly, debilitating constitutional symptoms and significant cytopaenias [1]. While therapeutic options have expanded significantly in the contemporary era, allogeneic haematopoietic cell transplantation (allo-HCT) remains the only potentially curative intervention [2]. Recently updated European Society for Blood and Marrow Transplantation (EBMT) and European LeukemiaNet (ELN; EBMT-ELN) consensus recommendations advocate to consider transplantation for primary MF patients with intermediate-II or high-risk disease as per the Dynamic International Prognostic Scoring System (DIPSS) or deemed high risk by the Mutation-Enhanced International Prognostic Score Systems (MIPSS70 or MIPSS70-plus) score paralleled by a low-intermediate risk Myelofibrosis Transplant Scoring System score (MTSS) [2]. For secondary MF (SMF), consideration is given for those with intermediate or high risk disease as per a validated SMF prognostic score. Individualised consideration was based upon patient and disease characteristics for those with DIPSS Intermediate-1 or MIPSS70/MIPSS70plus Intermediate with low-intermediate MTSS. In general, consideration was reserved for those up to the age of 70 with suitable donors and patients older than 70 years could be offered allo-HCT on an individual basis [2].

Recently, the treatment landscape has evolved dramatically with the approval of multiple JAK inhibitors (JAKi) across both the European Union (EU) and United State of America (USA). Ruxolitinib (Novartis, Switzerland), approved in 2012 by the European Medicine Agency, was followed by approvals for Fedratinib (Bristol Myers Squibb, UK; 2021), and subsequently Momelotinib (GlaxoSmithKline, UK; 2023) [3]. Of note, pacritinib is not available outside the clinical trial setting in the EU at the time of writing this manuscript [4]. The more novel JAKi, momelotinib and pacritinib, are not only effective at addressing constitutional symptoms and splenomegaly, but can also more specifically target disease-related anaemia, which remains a major source of morbidity and a marker of overall poor prognosis [5–8]. Furthermore, promising investigational therapies are in development, including telomerase inhibitors, BET inhibitors (BETi), BCL2 inhibitors (BCL2i), next-generation JAK inhibitors, and targeted therapies for mutated calreticulin (CALR), to name a few, which are making the therapeutic landscape and decision making even more complex [9–14].

Due to the increasing access to commercially available JAKi, a patient with MF may receive multiple lines of JAKi therapy prior to undergoing allo-HCT to control disease-related symptoms or splenomegaly. However, we know that responses to these agents are frequently heterogeneous, the median duration of clinically relevant response to ruxolitinib for example has been estimated at ~3-years, and true disease-modification is rare [15–17]. Paradoxically, the increasing availability of sequencing JAKi for those patients remaining potential allo-HCT MF candidates creates potential risks if we focus on long term survival: non-transplant focused physicians may choose to cycle through commercial drugs rather than refer to allo-HCT centres to facilitate early assessment and donor identification, disease progression or clonal evolution may occur during JAKi therapy, potentially worsening transplant outcomes, and the patient may actually become ineligible for transplant or have worse outcomes due to advancing age or frailty [18]. Previous work has highlighted substantial heterogeneity in transplant timing decisions, even prior to the widespread availability of these newer agents [19, 20].

Still in 2025, the management of patients transitioning from JAK inhibitors to allo-HCT remains highly varied across centres. Many centres employ weaning regimens due to concerns over abrupt withdrawal, although recommendations remain controversial, whereas others continue lower doses up until the time of engraftment or longer as maintenance strategies [21, 22]. The role of ruxolitinib for the management of steroid refractory or dependent graft-versus-host disease (GvHD) has become well established, raising questions regarding its continued use in MF patients post-transplant [23]. How best to use fedratinib, momelotinib and pacritinib peri- and indeed post- allo-HCT for MF remains unknown, and if different or similar strategies to ruxolitinib management should be employed.

Given these real-world complexities, we conducted a survey to capture current transplant practices for MF allo-HCT across multiple European centres. Specifically, we aimed to assess individual centres approaches to integration of JAKi into transplant protocols, approaches to managing splenomegaly prior to allo-HCT, timing of transplant and the influence of newer agents on the transplant decision-making process. By collecting real-world data from experienced transplant centres, this study seeks to identify emerging areas of both consensus and variation in practice. The results will directly inform future EBMT guideline updates, with the goal of improving and standardizing care for this complex allo-HCT patient population.

## Methods

The survey was developed by the Myeloproliferative Neoplasms (MPN) subcommittee of the EBMT Chronic Malignancies Working Party (CMWP). An electronic questionnaire was developed and distributed in SurveyMonkey by the EBMT data management team to 33 transplant centres across Europe. Eligible centres were selected based on their annual transplant activity, specifically those performing ≥5 MF allo-HCT procedures per year. The survey remained open for an eight-week period, during which two follow-up reminder emails were sent to encourage participation. By the time the survey closed, 19 centres had submitted responses. For each survey item, the term “total answers” refers to the number of valid responses received, with the corresponding percentage of all participating centres (*n* = 19) indicated in parentheses. Subsequent breakdowns are presented as absolute numbers along with their percentage of the total valid responses for that specific question.

## Results

A total of 19 transplant centres participated in the survey, all experienced in MF allo-HCT management. Details concerning location and transplant activity in relation to the average number of allo-HCT for MF per annum are highlighted in Table 1.

**Table 1:** Overview of transplant centres.

Country	Centre	Number of MF allo-HCT per year
France	Gustave Roussy Cancer Campus	6–10
	CHU Hotel-Dieu	1–5
	CHU Lille	6–10
	Nancy Hospital	6–10
	Saint-Louis	11–15
Germany	University Hospital Essen	11–15
	TUM University Hospital	11–15
	University Hospital Dresden	6–10
	University Hospital Ulm	11–15
Italy	ASST Papa Giovanni XXIII	6–10
	Spedali Civili di Brescia	11–15
	Fondazione Policlinico Universitario A. Gemelli-IRCCS	11–15
Netherlands	Amsterdam UMC	11–15
	UMC Utrecht	6–10
United Kingdom	University College London Hospitals	16–20
Poland	University Clinical Centre / Medical University of Gdańsk	6–10
Finland	Helsinki University Hospital	1–5
Norway	Oslo University Hospital	6–10
Switzerland	Basel university hospital	11–15

### Focus on utilisation of JAKi prior to allo-HCT

Centres were surveyed on the routine use of JAKi prior to allo-HCT for MF. The majority of centres (*n* = 13; 68%) reported routinely commencing JAKi for each newly diagnosed transplant-eligible patient prior to proceeding to transplant. Four centres (21%) indicated that the decision was based on specific clinical factors, while 2 centres (11%) reported not routinely using JAKi prior to starting conditioning. Despite the variation in criteria guiding treatment initiation, the underlying rationale was broadly consistent across centres: a reduction in splenomegaly and symptom control were the most frequently mentioned objectives. Once optimal response was achieved, nearly half of the centres (*n* = 9; 47%) reported routinely proceeding to transplant, rather than delaying. The remaining centres (53%) indicated that the actual decision to proceed was influenced by both patient characteristics and/or individual preference.

### Focus on splenomegaly management prior to Allo-HCT

**Fig. 1:**
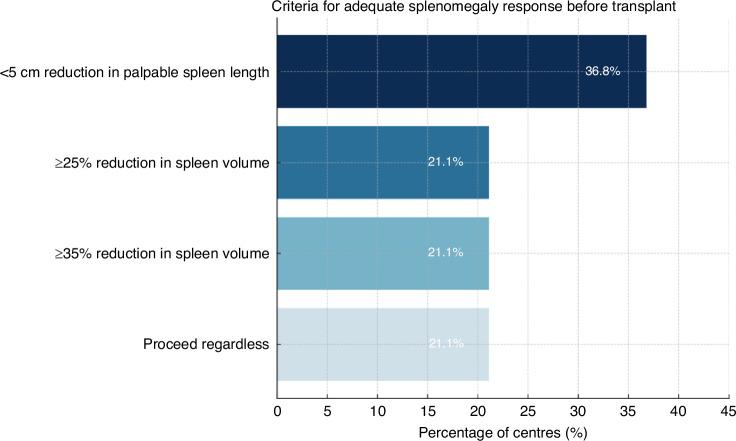
Criteria used by transplant centres to define adequate splenomegaly response prior to allogeneic haematopoietic stem cell transplantation. The most common criterion was a <5 cm reduction in palpable spleen length (36.8% of centres), followed by ≥25% reduction in spleen volume (21.1%), ≥35% reduction in spleen volume (21.1%), and proceeding regardless of spleen size (21.1%). Percentages represent the proportion of surveyed centres selecting each criterion.

Participating centres were surveyed regarding their utility of the recent EBMT position paper (Polverelli et al, 2023) on the optimal management of splenomegaly prior to allo-HCT [24]. All but one centre reported adherence, in general, to these recommendations. In the context of patients presenting with significant splenomegaly (defined as ≥5 cm below the left costal margin in the EBMT paper), centres were asked what degree of reduction would constitute an ‘adequate response’ prior to proceeding to allo-HCT. A reduction to <5 cm in palpable splenomegaly was considered sufficient by 7 centres (37%). Other centres preferred to assess spleen response using CT or MRI, considering a reduction of ≥25% spleen volume as acceptable in 4 centres (21%), while another 4 centres (21%) selected a threshold of ≥35% reduction in spleen volume. Of note, the remaining centres (*n* = 4; 21%) stated that they would proceed regardless of spleen response (Fig. 1).

### Focus of management of JAKi intolerance/resistance

**Fig. 2:**
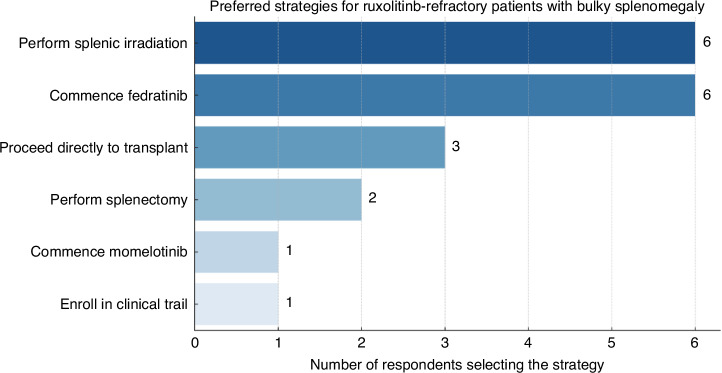
Preferred strategies for ruxolitinib-refractory patients with bulky splenomegaly. The most preferred strategy was to perform splenic irradiation (*n* = 6) and to commence Fedratinib (*n* = 6), followed by proceeding directly to a transplant (*n* = 3), splenectomy (*n* = 2), commencing Momelotinib (*n* = 1) and enrolment into a clinical trial (*n* = 1).

Centres were next asked specifically about the management of patients displaying ruxolitinib intolerance/resistance prior to allo-HCT. Approaches varied across the 19 responding centres, without any clear geographical pattern, and likely reflective of local experience and drug availability. Eight centres (42%) indicated they would switch to another JAKi (6 to fedratinib and 2 to momelotinib). In contrast, 7 centres (37%) would directly proceed to allo-HCT in this setting without sequencing to another JAKi or novel agent. Among the remaining 4 centres, two favoured enrolment into a clinical trial, while the other 2 would individualise the decision guided by specifics such as disease characteristics and donor availability. In cases of refractoriness to ruxolitinib and ongoing bulky splenomegaly, centres were asked to select six management strategies in order of preference. Splenic irradiation (*n* = 6) and commencing fedratinib (*n* = 6) were the preferred options, followed by proceeding directly to transplant (*n* = 3), splenectomy (*n* = 2), while commencing momelotinib and enroling in a clinical trial (*n* = 2) were selected by 1 centre (Fig. 2).

Centres were also surveyed regarding their approach once a second-line agent had been initiated or splenic irradiation performed. Specifically, they were asked whether they would proceed to transplant once clinician deemed ‘best response’ had been achieved. All but one centre reported that they would proceed to transplant at that point. The remaining centre chose to wait for ‘loss of response’ to 2nd line treatment to proceed to allo-HCT. Lastly, centres were asked to identify the most important patient characteristic that would prompt them to proceed to transplant without delay, rather than awaiting loss of response to current treatment. Nearly all centres (*n* = 16; 84%) cited the presence of high risk disease, either high DIPSS/DIPSS+ score or high risk mutational profile, as the principal driver for early transplant. The remaining three centres indicated that their decision was based on evidence suggesting improved outcomes with early transplantation compared to delayed approaches.

### Focus on Peri-allo-HCT management of JAKi and novel agents

#### JAKi management

**Fig. 3:**
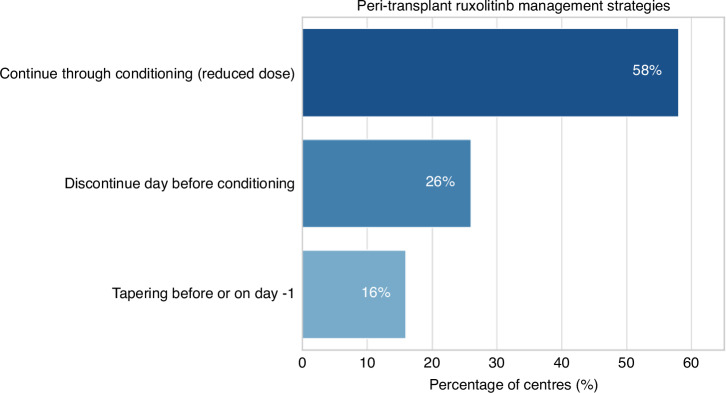
Peri-transplant ruxolitinib management strategies. Majority of centres (58%) would continue throughout conditioning at a reduced dose. 26% of centres would discontinue the day before conditioning and 16% will taper before or on one day prior to stem cell infusion.

Centres were surveyed regarding their peri-transplant management of ruxolitinib, fedratinib, momelotinib and pacritinib (if experienced with use in clinical trials prior to allo-HCT). When specifically asked, most centres chose ruxolitinib as their preferred JAKi (*n* = 15; 79%). Only 3 centres (16%) stated that they will choose the JAKi based on specific patient features. For ruxolitinib, the most frequent approach, reported by 11 centres (58%), was to continue the drug throughout conditioning, most commonly at a reduced dose, with timing of discontinuation variably occurring at the end of conditioning, at time of engraftment, or by day +21 post stem cell return. A total of 5 centres (26%) opted to discontinue ruxolitinib completely the day prior to conditioning. The remaining 3 centres (16%) adopted a tapering strategy, with drug cessation occurring either the day before conditioning starts or on day −1 (prior to stem cell return) (Fig. 3).

Regarding fedratinib, 5 centres (26%) reported no prior experience with the agent in the pre-transplant setting. Among those with experience, 5 centres (26%) opted to continue treatment throughout conditioning until engraftment, either at a reduced dose (3/5), or maximum tolerated dose (2/5). Another 5 centres (26%) reported stopping fedratinib the day before conditioning, while 4 centres (21%) adopted a tapering strategy for fedratinib prior to transplant.

Experience with momelotinib in the pre-transplant setting was more limited, with a total of 9 centres (47%) reporting no prior experience of use prior to allo-HCT. Amongst the centres with experience, 5 centres (26%) discontinued the treatment before the start of conditioning, with one stopping it on day −1, mirroring their particular approach with ruxolitinib. Two centres (11%) adopted a tapering strategy, while the other 3 (16%) continued treatment throughout conditioning, either at a reduced or maximum tolerated dose. Clinical experience with pacritinib in the pre-transplant setting was, as expected due to availability in the clinical trial setting only, also notably limited, with 14 centres (74%) reporting no experience. Of the remaining centres, 3 (16%) opted to continue pacritinib throughout conditioning and 2 centres (11%) reported stopping completely prior to the start of conditioning.

Lastly, centres were asked to describe real world challenges or complications encountered with the use of JAKi in the peri-transplant setting. Only 5 centres (26%) reported concerns in this context. Two centres documented cytopenia attributable to JAKi therapy. The remaining 3 centres each suggested a distinct complication, including infections, potential cardiotoxicity, and one case of Post Transplant Lymphoproliferative Disorder (PTLD), all of which clearly may be multifactorial in nature as regards risk.

#### Management of novel agents prior to allo-HCT

Centres were next surveyed regarding their experience and management strategies concerning novel agents such as BETi and BCL2 inhibitors prior to MF allo-HCT. Only 4 centres (21%) reported clinical experience in the use of BETi in the pre-transplant setting. Among these, the more common approach (*n* = 2) was to discontinue treatment the day before the start of conditioning. Of the remaining centres, one continued BETi throughout conditioning, while the other ceased treatment two weeks prior to transplant in order to minimise the risk of unexpected adverse effects. Experience with BCL2i (specifically Navitoclax, Abbvie) was similarly limited, with only 5 (26%) centres reporting experience with this agent prior to transplant, and the discontinuation strategies peri-transplant were heterogeneous. As with BETi discontinuation, the day before conditioning was the most common approach (*n* = 2). The remaining centres each reported a distinct strategy: one adopted a tapering approach, another continued treatment through conditioning, and the other one discontinued it two weeks before transplant to reduce potential toxicity. Lastly, we asked about experience transplanting patients who had previously been enroled in at least one clinical trial. While a few centres reported transplanting up to 12 such patients, the majority had not transplanted any, reflecting considerable variability in trial availability, participation across centres and networks and the numbers of patients moving ‘off’ clinical trials into the allo-HCT pathway. In this survey, the specific trials where patients had been enroled prior to allo-HCT included ADORE (ruxolitinib + siremadlin/crinalizumab/sabatolimab, NCT04097821), HOVON-134 (pacritinib, NCT03645824), LIMBER (BET inhibitor INC057643, NCT04551066), MANIFEST and MANIFEST-2 (pelabresib, NCT02158858, NCT04603495), PACIFICA (pacritinib, NCT03165734), and REFINE (navitoclax ± Ruxolitinib, NCT03222609). Despite this diversity of these investigational approaches, overall numbers remained small.

### Post-transplant management strategies

**Fig. 4:**
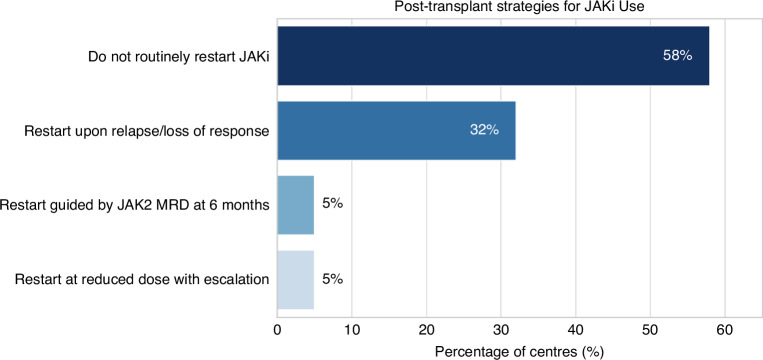
Post transplant strategies for JAK inhibitors (JAKi). The most common answer was no to routinely restart JAKi (58%), followed by restart only upon relapse or loss or response (32%). Only 5% of centres said that restart is guided by JAK2 MRD at 6 months or to restart routinely.

Centres were next surveyed regarding their approach to the use of JAKi after transplant. The majority (*n* = 11; 58%) reported that they do not routinely restart JAKi therapy. Of the remaining centres, 6 (32%) indicated that reintroduction would be considered in the event of loss of response or clear evidence of relapse. Of interest, 1 centre reported that the decision to restart was guided by the presence or absence of *JAK2* V617F or other MPN driver measurable residual disease (MRD) at a time point of six months post allo-HCT. Only one centre described a routine practice of reintroducing JAKi at a reduced dose post-transplant, with plans for gradual dose escalation, as a maintenance approach. Regarding patient-specific factors related to this decision, comorbidities and molecular risk profile were the 2 most common answers, with age and the stage of the disease at time of allo-HCT also being considered relevant. Centres were next asked to share their perceptions regarding potential benefits of JAKi use in the post-transplant setting. The most frequently perceived benefit was related to GVHD incidence reduction, with 5 centres (26%) reporting anecdotal experience of a suggested reduced incidence of acute GVHD and 4 centres (21%) noting a reduction in chronic GVHD. Two centres (11%) felt a more rapid reduction in spleen size occurred whereas none of the responding centres had the impression that JAKi reintroduction led to enhanced marrow fibrosis resolution (Fig. 4). Other treatment strategies for GvHD prophylaxis weren’t altered as a result of Ruxolitinib use.

#### Transplant outcomes and timing

The survey included questions exploring the perceived impact of JAKi on transplant timing and outcomes. Most of the centres (*n* = 15; 79%) reported a perceived positive impact on outcomes. When explored in more detail, more than half of the centres (*n* = 10, 53%) indicated a belief that use of these agents prior to allo-HCT was associated with an improved Event Free Survival (EFS). Reduction in relapse and GVHD incidence, as mentioned before, were also subjectively associated with use of JAKi prior to allo-HCT, being reported by 7 centres (37%) regarding reductions in GVHD and 5 centres (26%) regarding lower relapse rates. Focusing on timing of allo-HCT for patients on JAKi and/or novel agents, centres were specifically asked about perceived transplant delays. Nine centres (47%) expressed no concerns on this, not only regarding ruxolitinib but with other JAKi or even other novel therapies (BETi or BCL2i). However, several centres did report delays on entering allo-HCT, either regarding delayed referral for transplant consideration (*n* = 7; 37%) or a delay in then proceeding to transplant (*n* = 6; 32%). Finally, 11 centres (58%) reported cases in which patients initially deemed eligible for transplant progressed to a more aggressive disease phase as a consequence of transplantation delays incurred while undergoing initial medical therapy. This is obviously of major concern for transplant- eligible patients.

### Real world case scenarios

The survey included 3 real-world inspired cases to explore potentially differing clinical approaches by clinicians.

*Case 1*

*A 59-year old female patient has newly diagnosed JAK2 V617F mutated DIPSS-plus high risk MF with massive splenomegaly. She has an HLA-identical brother as a potential donor. She is otherwise healthy. Her bone marrow biopsy identifies fibrosis grade 3 and a blast count of 5%. She carries TP53 and ASXL1 pathogenetic mutations. Karnofsky performance status is 100%. MTSS score is calculated as very high. How would you proceed?*

Results: All but one centre reported an approach of commencing ruxolitinib and planning for transplant once the donor was available and pre-transplant screen completed. The remaining centre indicated that they would proceed directly to transplant planning, without use of JAKi, and refer the patient for either splenectomy or splenic radiotherapy.

*Case 2*

*A 64-years male patient with newly diagnosed DIPSS-plus Intermediate-2 CALR mutated MF and transfusion dependence. He has a normal sized spleen. He has a matched unrelated donor identified. Karnofsky performance status is 80%. MTSS score is intermediate. How would you proceed?*

Results: Most centres (*n* = 14; 74%) reported a strategy of commencing ruxolitinib and planning transplant once a donor was available and pre-transplant evaluation completed. The remaining centres (*n* = 5; 26%) preferred a ‘watch-and-wait approach’ with no JAKi use while planning transplant.

*Case 3*

*A 63-years female patient with DIPSS-plus high risk JAK2 V617F mutated MF with splenomegaly has been progressing on ruxolitinib (cytopenia and transfusion dependence). She has an HLA-matched sister as a potential donor. Karnofsky performance status is 100%. MTSS score is intermediate. How would you proceed?*

Results: In this case, all centres indicated they would proceed to transplant; however, their approaches to pre-transplant management were varied. Eleven centres (58%) reported ideally initiating a second-line therapy prior to transplant. Of these, nine centres (47%) would commence fedratinib or another experimental agent, while two centres (11%) preferred momelotinib. The remaining eight centres (42%) would proceed directly to transplant, either through an observant approach direct to allo-HCT (*n* = 4; 21%) or following splenectomy or splenic radiotherapy (*n* = 3; 16%).

## Discussion

This EBMT-CMWP survey reveals ongoing heterogeneity in the transplant decision-making process for MF allo-HCT, timing of transplant and peri-transplantation practices across European centres, as the arena of medical therapies expands. The survey underscores the reality of several potential ongoing areas of potential ‘disconnect’ between transplant centres and referring MPN-treating physicians goals. Clearly, there are many factors that play into these real-world decisions, including functional relationships between MPN- and allo-HCT focused clinicians and establishments of networks, historical centre practice, availability of commercial agents and extent of clinical trial portfolio, clinician and, most importantly, patient perceptions and goals.

One of the most clinically relevant findings is that approximately half of the centres preferred early allo-HCT in eligible patients, at the time of best response to therapy, despite the increasing availability of commercial JAKi and novel therapeutic agents. However, we acknowledge that the survey included transplant centres who may be biased towards transplant and that this would not capture potential allo-HCT candidates who have not been referred to allo-HCT units. Additionally, due to our selection criteria, only experienced MPN transplant centres were included, while we had a return rate of 19/33 which may limit the generalisation of our results. Within these limitations, most centres continued to favour early referral and rapid transition to a transplant pathway once a suitable donor is identified, particularly in the context of high-risk disease/ adverse molecular features. While nearly all centres incorporated the use of JAK inhibitors, primarily ruxolitinib, into their pre-transplant strategy, the overarching goal of such therapies remains symptom control and splenic reduction prior to definitive curative treatment with allo-HCT. Of note, just over half of centres surveyed said the timing of allo-HCT, in contrast, was decided on clinical factors and patient preference. Centre-specific factors, such as the cut-off age limit for MF transplant may also influence these decisions. The survey did not highlight any particular preference for delay in transplant decision in favour of prolonged exposure to novel agents, even among centres familiar with newer agents like fedratinib, momelotinib, or experimental drugs such as BETi or BCL2i, but clearly guidance is needed for such patients as regards optimal transplant timing.

This emphasis on early transplant runs counter to what might be expected given the broader MPN treatment paradigm, where novel agents are increasingly used in sequence and often for prolonged periods by non-transplant focused clinicians. The survey highlights a clear, and ongoing, disconnect whereby transplant centres frequently report delays in referrals or in transplant scheduling attributable to ongoing medical therapies initiated by non-transplant focussed haematologists. Indeed, worryingly, over half of the centres reported cases where patients progressed to more aggressive disease phases while undergoing medical therapies. This suggests a potential misalignment in risk assessment and therapeutic urgency between transplant and non-transplant teams and highlights the ongoing issue that many potential allo-HCT candidates never actually make it to transplant. Better network communication and wider participant tumour boards may help smoothen these pathways for MF patients. We acknowledge that a survey of non-transplant physicians on these topics is essential to understand further decision processes and practice.

It was reassuring to note that all but 1 centre reported general adherence to the EBMT position paper on the optimal management of splenomegaly prior to allo-HCT [24]. There remains heterogeneous practice, however, with regards management of allo-HCT candidates who have either ruxolitinib intolerance/ resistance with some centres proceeding directly to allo-HCT and others exploring other JAKi or therapeutic strategies. As a community, efforts should be co-ordinated here to understand the best approach for improving not only survival post allo-HCT as the main goal but also amelioration in poor graft function and GVHD incidence/ relapse risk. A clinical trial is required but would need to be a large, multinational style trial to understand the best approach.

Retrospective data from the EBMT highlighted the potential of improved EFS for patients undergoing allo-HCT at the time of best response to ruxolitinib, alongside a trend towards less relapse, compared to patients who underwent allo-HCT and were JAKi naïve or had lost/ never gained a response to ruxolitinib [25].Ali and colleagues reported on peri-transplantation use of ruxolitinib in a small, prospective open label study, highlighting safety, no delays in engraftment and low rates of higher grades of acute GVHD [26]. Our study reveals heterogeneous practice as regards to best timing of allo-HCT for patients on JAKi and this will need to be addressed. Moreover, prospective trials evaluating the risk/ benefit ration of peri-transplant use of the more novel agents pacritinib and momelotinib are required, including an understanding of any potential relapse or GVHD risk modification. The survey also highlights heterogenous approaches in how transplant centres manage comparable clinical scenarios (within the 3 case studies) likely due to lack of convincing evidence for the perceived ‘correct’ approach, centre experience and the relatively low numbers of procedures performed per centre given the rarity of MF as an allo-HCT indication. For instance, while nearly all centres would initiate JAK inhibitor therapy in high-risk MF with an available donor, opinions diverged on whether to use bridging therapy versus proceeding directly to transplant in JAKi-refractory patients. Duration of JAKi prior to transplant is also another important element that may rely on the timing of the referral to the transplant centre. Similarly, management approaches for patients progressing on ruxolitinib varied from immediate transplant to second-line agent use or trial enrolment, reflecting a lack of standardized algorithms. Even among centres with access to more recently available JAKi, their practical integration into peri-transplant protocols varied widely, particularly regarding tapering schedules, dosing during conditioning, and post-transplant reintroduction. Real world safety data on all of these approaches is required on a collaborative basis, particularly looking into amelioration of GvHD as well as partient specific factors which may affect outcomes.

Without consensus on optimal timing and integration of therapies, patient outcomes are likely to remain variable. This is a challenge that would likely be heightened in view of more upcoming and potentially disease modifying treatments such as *CALR* mutant directed immunotherapies. Use of novel agents is still exploratory, and post-transplant strategies appear centre-dependent and evolving. We need to learn more about how we can harness the potential effects of BCL2, BETi and other agents on improving MF allo-HCT outcomes, on a wider perspective than spleen and symptom control prior. For example, BET domain inhibition has been shown to reduce acute GVHD in murine models and BCL2 has been suggested as an important factor for GVHD development [27, 28].

## Conclusion

Our findings point to a need for harmonized, multidisciplinary guidelines that bridge the gap between MPN-treating physicians and transplant centres. Enhanced collaboration, ideally commencing at the time of diagnosis, will be essential to optimize timing, leverage novel therapies appropriately, and ensure that transplant-eligible patients are not exposed to undue risks from delayed curative intervention. Additionally, it is important that patients are informed about available trials while we are eagerly expecting more data about emerging therapies that may further enhance the current treatment paradigm and become integrated into the transplant pathway.

## Data Availability

All data obtained from this study are included in this manuscript. Raw data can be requested via contacting the EBMT Leiden study unit.

## References

[1] McLornan DP, Psaila B, Ewing J, Innes A, Arami S, Brady J, et al. The management of myelofibrosis: a British Society for Haematology Guideline. Br J Haematol. 2024;204:136–50. 10.1111/bjh.19186. 38037886 10.1111/bjh.19186

[2] Kröger N, Bacigalupo A, Barbui T, Ditschkowski M, Gagelmann N, Griesshammer M, et al. Indication and management of allogeneic haematopoietic stem-cell transplantation in myelofibrosis: updated recommendations by the EBMT/ELN International Working Group. Lancet Haematol. 2024;11:e62–74. 10.1016/S2352-3026(23)00305-8. 38061384 10.1016/S2352-3026(23)00305-8

[3] Masarova L, Chifotides HT. How I individualize selection of JAK inhibitors for patients with myelofibrosis. Blood. 2025;145:1724–37. 10.1182/blood.2023022415. 39357058 10.1182/blood.2023022415 PMC12060163

[4] Mascarenhas J, Gerds AT, Kiladjian J-J, Döhner K, Buckley S, Smith JA, et al. PACIFICA: a randomized, controlled phase 3 study of pacritinib versus physician’s choice in patients with primary or secondary myelofibrosis and severe thrombocytopenia. Blood. 2022;140:9592–4. 10.1182/blood-2022-163456.

[5] Harrison CN, Vannucchi AM, Platzbecker U, Cervantes F, Gupta V, Lavie D, et al. Momelotinib versus best available therapy in patients with myelofibrosis previously treated with ruxolitinib (SIMPLIFY 2): a randomised, open-label, phase 3 trial. Lancet Haematol. 2018. 10.1016/S2352-3026(17)30237-5. 10.1016/S2352-3026(17)30237-5 29275119

[6] Mesa RA, Kiladjian J-J, Catalano JV, Devos T, Egyed M, Hellmann A, et al. SIMPLIFY-1: a phase III randomized trial of momelotinib versus ruxolitinib in Janus kinase inhibitor-naïve patients with myelofibrosis. J Clin Oncol J Am Soc Clin Oncol. 2017;35:3844–50. 10.1200/JCO.2017.73.4418. 10.1200/JCO.2017.73.4418 PMC6553796 28930494

[7] Verstovsek S, Gerds AT, Vannucchi AM, Al-Ali HK, Lavie D, Kuykendall AT, et al. Momelotinib versus danazol in symptomatic patients with anaemia and myelofibrosis (MOMENTUM): results from an international, double-blind, randomised, controlled, phase 3 study. Lancet. 2023;401:269–80. 10.1016/S0140-6736(22)02036-0. 36709073 10.1016/S0140-6736(22)02036-0

[8] Mesa RA, Vannucchi AM, Mead A, Egyed M, Szoke A, Suvorov A, et al. Pacritinib versus best available therapy for the treatment of myelofibrosis irrespective of baseline cytopenias (PERSIST-1): an international, randomised, phase 3 trial. Lancet Haematol. 2017;4:e225–36. 10.1016/S2352-3026(17)30027-3. 28336242 10.1016/S2352-3026(17)30027-3 PMC8209752

[9] Ma W, Balaian L, Mondala P, He Y, Mason C, Pham J, et al. Imetelstat inhibits telomerase and prevents propagation of ADAR1-activated myeloproliferative neoplasm and leukemia stem cells. Blood. 2020;136:18–18. 10.1182/blood-2020-140771.

[10] Harrison CN, Garcia JS, Mesa RA, Somervaille TC, Komrokji RS, Pemmaraju N, et al. Results from a phase 2 study of navitoclax in combination with ruxolitinib in patients with primary or secondary myelofibrosis. Blood. 2019;134:671–671. 10.1182/blood-2019-130158.

[11] Mascarenhas J, Harrison C, Luptakova K, Christo J, Wang J, Mertz JA, et al. MANIFEST-2, a global, phase 3, randomized, double-blind, active-control study of CPI-0610 and ruxolitinib Vs. placebo and ruxolitinib in JAK-inhibitor-naive myelofibrosis patients. Blood. 2020;136:43. 10.1182/blood-2020-140901.

[12] Mascarenhas J, Kremyanskaya M, Patriarca A, Palandri F, Devos T, Passamonti F, et al. MANIFEST: pelabresib in combination with ruxolitinib for Janus kinase inhibitor treatment-naïve myelofibrosis. J Clin Oncol 2023:JCO.22.01972. 10.1200/JCO.22.01972. 10.1200/JCO.22.01972 PMC10642902 36881782

[13] Rampotas A, Wong Z, Gannon I, Benlabiod C, Shen Y, Brierley C, et al. Development of a first-in-class CAR-T therapy against calreticulin-mutant neoplasms and evaluation in the relevant human tissue environment. Blood. 2024;144:871–871. 10.1182/blood-2024-204953.

[14] Mascarenhas JO, Borate U, Bose P, Byrd JC, Garcia JS, Grunwald MR, et al. A multicenter, open-label, phase 1 clinical trial of AJ1-11095 administered as oral monotherapy in patients with primary myelofibrosis (PMF), post-polycythemia vera myelofibrosis (PPV-MF), or post-essential thrombocythemia myelofibrosis (PET-MF) who have been failed by a type I JAK2 inhibitor (JAK2i). Blood. 2024;144:3147.1–3147.1. 10.1182/blood-2024-193905.

[15] Palandri F, Breccia M, Bonifacio M, Polverelli N, Elli EM, Benevolo G, et al. Life after ruxolitinib: Reasons for discontinuation, impact of disease phase, and outcomes in 218 patients with myelofibrosis. Cancer. 2020;126:1243–52. 10.1002/cncr.32664. 31860137 10.1002/cncr.32664

[16] Palandri F, Breccia M, Mazzoni C, Auteri G, Elli EM, Trawinska MM, et al. Ruxolitinib in cytopenic myelofibrosis: Response, toxicity, drug discontinuation, and outcome. Cancer. 2023;129:1704–13. 10.1002/cncr.34722. 36932983 10.1002/cncr.34722

[17] Palandri F, Palumbo GA, Elli EM, Polverelli N, Benevolo G, Martino B, et al. Ruxolitinib discontinuation syndrome: incidence, risk factors, and management in 251 patients with myelofibrosis. Blood Cancer J. 2021;11:4. 10.1038/s41408-020-00392-1. 33414394 10.1038/s41408-020-00392-1 PMC7791065

[18] McLornan D, Szydlo R, Koster L, Chalandon Y, Robin M, Wolschke C, et al. Myeloablative and Reduced-Intensity Conditioned Allogeneic Hematopoietic Stem Cell Transplantation in Myelofibrosis: A Retrospective Study by the Chronic Malignancies Working Party of the European Society for Blood and Marrow Transplantation. Biol Blood Marrow Transpl. 2019;25:2167–71. 10.1016/j.bbmt.2019.06.034. 10.1016/j.bbmt.2019.06.034 31284069

[19] Cipkar C, Kumar S, Thavorn K, Kekre N. Optimal timing of allogeneic stem cell transplantation for primary myelofibrosis. Transpl Cell Ther Publ Am Soc Transpl Cell Ther. 2022;28:189–94. 10.1016/j.jtct.2022.01.018. 10.1016/j.jtct.2022.01.018 35077904

[20] McLornan D, Eikema DJ, Czerw T, Kröger N, Koster L, Reinhardt HC, et al. Trends in allogeneic haematopoietic cell transplantation for myelofibrosis in Europe between 1995 and 2018: a CMWP of EBMT retrospective analysis. Bone Marrow Transpl. 2021;56:2160–72. 10.1038/s41409-021-01305-x. 10.1038/s41409-021-01305-x 33911203

[21] Rathje K, Gagelmann N, Badbaran A, Langebrake C, Dadkhah A, Richter J, et al. Clinical and immune effects of PERI-transplantation JAK inhibition for myelofibrosis. Am J Hematol. 2025;100:200–9. 10.1002/ajh.27529. 39548811 10.1002/ajh.27529 PMC11705205

[22] Villar S, Curis E, Schlageter M-H, Bosselut N, Charbonnier A, Rubio M-T, et al. Ruxolitinib stopped before transplantation does not induce cytokine release in myelofibrosis. Cancer Immunol Immunother CII. 2025;74:181 10.1007/s00262-025-04046-8. 40274649 10.1007/s00262-025-04046-8 PMC12022196

[23] Ruxolitinib for the treatment of acute and chronic graft-versus-host disease in children: a systematic review and individual patient data meta-analysis. Bone Marrow Transplant. 2024. https://www.nature.com/articles/s41409-024-02252-z. 10.1038/s41409-024-02252-z PMC11161405 38402346

[24] Polverelli N, Hernández-Boluda JC, Czerw T, Barbui T, D’Adda M, Deeg HJ, et al. Splenomegaly in patients with primary or secondary myelofibrosis who are candidates for allogeneic hematopoietic cell transplantation: a Position Paper on behalf of the Chronic Malignancies Working Party of the EBMT. Lancet Haematol. 2023;10:e59–70. 10.1016/S2352-3026(22)00330-1. 36493799 10.1016/S2352-3026(22)00330-1

[25] Kröger N, Sbianchi G, Sirait T, Wolschke C, Beelen D, Passweg J, et al. Impact of prior JAK-inhibitor therapy with ruxolitinib on outcome after allogeneic hematopoietic stem cell transplantation for myelofibrosis: a study of the CMWP of EBMT. Leukemia 2021. 10.1038/s41375-021-01276-4. 10.1038/s41375-021-01276-4 PMC8632691 34023851

[26] Ali H, Tsai N-C, Synold T, Mokhtari S, Tsia W, Palmer J, et al. Peritransplantation ruxolitinib administration is safe and effective in patients with myelofibrosis: a pilot open-label study. Blood Adv. 2022;6:1444–53. 10.1182/bloodadvances.2021005035. 34581764 10.1182/bloodadvances.2021005035 PMC8905711

[27] Snyder KJ, Choe HK, Gao Y, Sell NE, Braunreiter KM, Zitzer NC, et al. Inhibition of Bromodomain and Extra Terminal (BET) domain activity modulates the IL-23R/IL-17 axis and suppresses acute graft-versus-host disease. Front Oncol. 2021;11:760789. 10.3389/fonc.2021.760789. 34722316 10.3389/fonc.2021.760789 PMC8554203

[28] Strobl J, Pandey RV, Krausgruber T, Kleissl L, Reininger B, Herac M, et al. Anti-apoptotic molecule BCL2 is a therapeutic target in steroid-refractory graft-versus-host disease. J Invest Dermatol. 2020;140:2188–98. 10.1016/j.jid.2020.02.029. 32247860 10.1016/j.jid.2020.02.029

